# Altered pathways and colorectal cancer prognosis

**DOI:** 10.1186/s12916-015-0307-6

**Published:** 2015-04-08

**Authors:** Victor Moreno, Rebeca Sanz-Pamplona

**Affiliations:** Cancer Prevention and Control Program, Catalan Institute of Oncology (ICO), Bellvitge Biomedical Research Institute (IDIBELL) and Consortium for Biomedical Research in Epidemiology and Public Health (CIBEResp), Barcelona, Spain; Department of Clinical Sciences, Faculty of Medicine, University of Barcelona, Barcelona, Spain

**Keywords:** Colorectal cancer, Prognosis, Pathways, Molecular subtypes

## Abstract

The identification of molecular markers with prognostic value in colorectal cancer is a challenging task that is needed to define therapeutic guidelines. Clinical factors are insufficient to identify those patients with stage II at risk of relapse or those patients with stage III at low risk. There is a current effort to define a consensus in molecular subtypes based on expression profiles, which are characterized by a distinctive prognostic outcome. Also several gene expression signatures based on individual genes have been proposed to predict prognosis, but they show low consistency and reproducibility. Slattery et al. describe a pathway-based approach to analyze gene expression differences between normal and colon cancer tissues. The most interesting finding is that having more deregulated pathways is associated with good prognosis. If these findings are properly validated, new insights into the mechanisms of colon carcinogenesis may be revealed.

Please see related article: http://dx.doi.org/10.1186/s12916-015-0292-9.

## Background

Colorectal cancer (CRC) is a frequently occurring disease with high mortality in which prognosis is dramatically dependent on stage at diagnosis. Adjuvant chemotherapy is the current standard treatment for stage III but still controversial in stage II. Recognized clinical risk factors are insufficient to identify those patients with stage II at risk of relapse or those patients with stage III at low risk, leading to potential under or over-treatment [1]. Recently, multiple efforts have been devoted to characterize the complex molecular landscape of CRC, aiming to identify key genes involved in cancer development and prognosis [2]. This should provide therapeutic targets or prognostic biomarkers. Mutations in KRAS and TP53 genes are frequent, but lack prognostic value. The subgroup of tumors showing microsatellite instability (MSI) have better prognosis, but may be resistant to standard 5-fluorouracil adjuvant therapy. Mutations in BRAF, though less frequent, may define a subgroup of poor prognosis. Finally, another subgroup of tumors show the CpG island methylator phenotype (CIMP). This group overlaps with the MSI and BRAF mutated groups and does not have a clear independent prognostic value.

Recently, whole genome analyses of tumors have tried to tackle this topic, either with the analysis of microarrays or, more recently, with RNA-seq expression analysis. The efforts have followed two approaches, either to define prognostic signatures based on a subset of genes or to define molecular subtypes, identified with unsupervised modeling strategies, which in a second step are characterized clinically and attributed a prognostic value [3]. There is a current effort to define a consensus molecular subtypes classification, since different studies have identified diverse numbers of subtypes and genes related to these subtypes [4]. Similarly, several prognostic profiles based on gene expression have been proposed, with variable predictive ability and not always with proper validation, which has detracted from their introduction in clinical practice [5]. Comparisons of these expression predictors usually show low overlap when specific genes are analyzed, and this has been interpreted to mean that the profiles are a random sample of a higher functional hierarchy that probably is related to a few functional pathways [6].

## Survival among cancer patients with increased differentially expressed pathways

Slattery et al. [7] describe in BMC Medicine a pathway-based approach to analyze whole genome expression changes in colon cancer tissues when compared to normal adjacent tissue samples. The main conclusion of the study is that having more deregulated pathways is associated with good prognosis [7]. This research is innovative in the analytical approach. The study initially compared RNA-seq expression data from 175 colon tumors with their paired adjacent normal mucosa. Differentially expressed genes found were then assigned to pathways, and the relevant explanatory variable analyzed was the number of deregulated genes within pathways. Using a simple method, each patient was assigned a deregulation score based on the number of altered genes for each pathway. Interestingly, they found that having a high score was associated with better survival in 16 pathways, after adjusting for age, stage, sex, and tumor molecular phenotype (MSI, TP53, KRAS, and CIMP). The most significant pathways involved functions related to cell signaling and growth.

The pathway approach used is an interesting strategy, since deregulation of different genes could converge in the same pathway. This allows tumors exhibiting dissimilar gene expression patterns to achieve a similar phenotype. In consequence, collapsing genes into pathways could be a useful tool not only to summarize a large list of genes into more comprehensive functional entities, but also to classify a priori molecularly different tumors into subtypes. From a translational point of view this categorization has a special relevance, since tumors exhibiting different gene expression patterns could behave in an analogous way regarding treatment response or patient outcome. Indeed, similar pathway-oriented approaches have been described to be more useful than those based on expression of individual genes [8] and reported to be informative about prognosis in breast cancer [9].

The authors of this study reported cell cycle as the most important pathway related to CRC prognosis along with metabolic pathways and others classically related to colon carcinogenesis like the Wnt pathway. Other studies on molecular subtypes using gene expression signatures have described “epithelial” tumors as highly proliferative and with better prognosis than tumors with a “mesenchymal” phenotype [10-12]. It is intriguing that the epithelial-to-mesenchymal transition (EMT) pathway did not appear as a relevant pathway in their analysis, which should be related to poor prognosis. However, the authors identified TGFB1 as an upstream regulator of genes showing differential expression using a network analysis. TGFB1 is a tumor suppressor gene regarding tumor initiation, but also induces EMT and acts as a promoter of metastasis [13,14]. This dual role of TGFB1 makes the interpretation of its function difficult, but EMT induced by TGFB1 expression usually has been related to poor prognosis [10,13-15]. Also, regarding the functional interpretation of the data of Slattery’s study, a limitation is that gene expression in normal tissue adjacent to the tumor has been shown to be altered, when compared to the expression in healthy subjects [16]. Probably there is a cross-talk between tumor and normal adjacent tissue in addition to the tumor micro-environment cross-talk, and many of the gene expression signals captured by RNA-seq derive from stroma and not from the tumor cell [17]. This does not deny the potential prognostic value of the differences, but it could modify the interpretation of some of the pathways’ effects and the strategy to design targeted therapies.

Another interesting finding revealed in Slattery’s study is that pathway deregulation is not only related to prognosis but is also inversely related to stage. Tumors from patients diagnosed in stage I had more differentially expressed genes than those from patients with advanced cancer, though the differences were not statistically significant. This finding, if confirmed in other studies, opens new questions related to CRC progression. Why do less advanced tumors have more differentially expressed genes? Are metastatic tumors more specialized than early stage ones and need less altered genes to survive once they have acquired metastatic potential? Large gene expression changes in cell cycle observed in stage I and II tumors could suggest that these tumors are able to proliferate and grow in size but are unable to develop more sophisticated functions necessary to invade and disseminate. As the authors propose, perhaps increased gene expression changes could be destabilizing for the tumor and, in consequence, responsible for better prognosis.

Despite the interesting findings reported, a major limitation of the study is that the authors have not been able to provide a validation of their results in an independent dataset. There are available public datasets on colon cancer expression that simultaneously have analyzed tumor and paired mucosa that can be used to validate differential expression genes [18], but this dataset is restricted to stage II tumors and has not published data on prognosis yet. Other interesting datasets like The Cancer Genome Atlas (TCGA) consortium study [19] have analyzed very few normal tissues, and this hinders its utility to validate results that focus on differences between tumor and normal tissue such as the specific alterations observed in Slattery’s study.

## Conclusions

In conclusion, this work by Slattery et al. reports a novel pathway-based approach to predict survival in CRC patients. This novel strategy could overcome gene expression signature approaches. However, although promising, these findings need further validation. If the prognostic value of these pathway scores can be replicated in independent datasets, the findings would represent a major contribution and warrant a novel view in understanding gene deregulation observed in colon cancer.

## Abbreviations

CRC: colorectal cancer
CIMP: CpG island methylator phenotype
EMT: epithelial-to-mesenchymal transition
MSI: microsatellite instability

## References

[1] Gill S, Loprinzi CL, Sargent DJ, Thome SD, Alberts SR, Haller DG (2004). Pooled analysis of fluorouracil-based adjuvant therapy for stage II and III colon cancer: who benefits and by how much?. J Clin Oncol.

[2] Fearon ER (2011). Molecular genetics of colorectal cancer. Annu Rev Pathol.

[3] Sanz-Pamplona R, Santos C, Grasselli J, Molleví DG, Dienstmann R, Paré-Brunet L (2014). Unsupervised analyses reveal molecular subtypes associated to prognosis and response to therapy in colorectal cancer. Colorectal Cancer.

[4] Sadanandam A, Wang X, de Sousa EMF, Gray JW, Vermeulen L, Hanahan D (2014). Reconciliation of classification systems defining molecular subtypes of colorectal cancer: interrelationships and clinical implications. Cell Cycle.

[5] Sanz-Pamplona R, Berenguer A, Cordero D, Riccadonna S, Sole X, Crous-Bou M (2012). Clinical value of prognosis gene expression signatures in colorectal cancer: a systematic review. PLoS One.

[6] Lascorz J, Chen B, Hemminki K, Forsti A (2011). Consensus pathways implicated in prognosis of colorectal cancer identified through systematic enrichment analysis of gene expression profiling studies. PLoS One.

[7] Slattery ML, Herrick JS, Mullany LE, Gertz J, Wolff RK. Improved survival among colon cancer patients with increased differentially expressed pathways. BMC Med. 2015. doi:10.1186/s12916-015-0292-9. 10.1186/s12916-015-0292-9 PMC4389992 25890236

[8] Borisov NM, Terekhanova NV, Aliper AM, Venkova LS, Smirnov PY, Roumiantsev S (2014). Signaling pathways activation profiles make better markers of cancer than expression of individual genes. Oncotarget.

[9] Huang S, Yee C, Ching T, Yu H, Garmire LX (2014). A novel model to combine clinical and pathway-based transcriptomic information for the prognosis prediction of breast cancer. PLoS Comput Biol.

[10] Loboda A, Nebozhyn MV, Watters JW, Buser CA, Shaw PM, Huang PS (2011). EMT is the dominant program in human colon cancer. BMC Med Genomics.

[11] Roepman P, Schlicker A, Tabernero J, Majewski I, Tian S, Moreno V (2014). Colorectal cancer intrinsic subtypes predict chemotherapy benefit, deficient mismatch repair and epithelial-to-mesenchymal transition. Int J Cancer.

[12] Schlicker A, Beran G, Chresta CM, McWalter G, Pritchard A, Weston S (2012). Subtypes of primary colorectal tumors correlate with response to targeted treatment in colorectal cell lines. BMC Med Genomics.

[13] Lamouille S, Xu J, Derynck R (2014). Molecular mechanisms of epithelial-mesenchymal transition. Nat Rev Mol Cell Biol.

[14] Miyazono K (2009). Transforming growth factor-beta signaling in epithelial-mesenchymal transition and progression of cancer. Proc Jpn Acad Ser B Phys Biol Sci.

[15] Bhangu A, Wood G, Mirnezami A, Darzi A, Tekkis P, Goldin R (2012). Epithelial mesenchymal transition in colorectal cancer: Seminal role in promoting disease progression and resistance to neoadjuvant therapy. Surg Oncol.

[16] Sanz-Pamplona R, Berenguer A, Cordero D, Mollevi DG, Crous-Bou M, Sole X (2014). Aberrant gene expression in mucosa adjacent to tumor reveals a molecular crosstalk in colon cancer. Mol Cancer.

[17] Isella C, Terrasi A, Bellomo SE, Petti C, Galatola G, Muratore A, et al. Stromal contribution to the colorectal cancer transcriptome. Nat Genet. 2015. doi:10.1038/ng.3224. 10.1038/ng.3224 25706627

[18] Cordero D, Sole X, Crous-Bou M, Sanz-Pamplona R, Pare-Brunet L, Guino E (2014). Large differences in global transcriptional regulatory programs of normal and tumor colon cells. BMC Cancer.

[19] Cancer Genome Atlas N. Comprehensive molecular characterization of human colon and rectal cancer. Nature. 2012;487:330–37. 10.1038/nature11252 PMC3401966 22810696

